# Sheep breed and shearing influences attraction and blood-feeding behaviour of *Culicoides* (Diptera: Ceratopogonidae) on a UK farm

**DOI:** 10.1186/s13071-018-3003-5

**Published:** 2018-08-20

**Authors:** Andrew Hope, Simon Gubbins, Christopher Sanders, James Barber, Francesca Stubbins, Matthew Baylis, Simon Carpenter

**Affiliations:** 1 The Pirbright Institute, Pirbright, Surrey UK; 2 Liverpool University Climate and Infectious Diseases of Animals (Lucinda) Group, University of Liverpool, Neston, Cheshire UK; 3 Health Protection Research Unit in Emerging and Zoonotic Infections, University of Liverpool, Liverpool, UK

**Keywords:** Bluetongue virus, Schmallenberg virus, Animal husbandry, Vector, Arbovirus, Host location

## Abstract

**Background:**

*Culicoides* biting midges (Diptera: Ceratopogonidae) are responsible for the biological transmission of arboviruses of international importance between ruminant livestock. These arboviruses include bluetongue virus (BTV) and Schmallenberg virus (SBV), which have emerged in unprecedented outbreaks in northern Europe. The impact of breed and shearing of sheep on *Culicoides*: host contact rates has not been investigated in detail and has the potential to influence arbovirus transmission and control measures employed to limit spread.

**Methods:**

Attraction of *Culicoides* to Hartline and Hartline/Suffolk cross-breed sheep was compared using 224 drop trap collections over 22 nights and 181 catches from sheared or unsheared Hartline/Suffolk ewes were made over 17 nights to compare *Culicoides* activity and rates of blood engorgement.

**Results:**

A total of 31,314 *Culicoides* was collected in the two trials and females of the subgenus *Avaritia* represented over 96.9% of individuals collected. Attraction to breed was dependent upon species of *Culicoides* and physiological status, with a significantly greater number of individuals collected on the cross-breed sheep. Shearing of sheep did not significantly increase or decrease the number of *Culicoides* attracted but increased the rate of successful engorgement.

**Conclusions:**

Both breed and shearing were shown to influence *Culicoides* biting rate on sheep. These data are useful in a direct context in understanding the likely impact of control measures against arboviruses including BTV and SBV and additionally in providing data from field-based studies to enable modelling exercises of arbovirus transmission and spread.

**Electronic supplementary material:**

The online version of this article (10.1186/s13071-018-3003-5) contains supplementary material, which is available to authorized users.

## Background

*Culicoides* biting midges (Diptera: Ceratopogonidae) are biological vectors of internationally important arboviruses [1, 2]. Bluetongue virus (BTV) and Schmallenberg virus (SBV) epidemics have inflicted major economic impact on farms across Europe, not only in causing direct clinical disease (most commonly in sheep), but also in leading to the imposition of animal movement restrictions to limit disease spread [3, 4]. Vaccination of susceptible livestock against these arboviruses is a highly effective tool in combating incursions in Europe and has been used several times successfully to eradicate BTV from countries [5, 6]. Use of vaccines does, however, require a substantial period of time to implement due to assessment of outbreak impact (which determines commercial production), development, testing and registration. Prior to the use of vaccines, the only control measures available to limit spread and impact of *Culicoides*-borne arboviruses are those aimed at reducing *Culicoides*, host contact [7–9] and ruminant movement restrictions.

It has long been suggested that sheep breeds vary in their susceptibility to clinical diseases caused by *Culicoides*-borne arboviruses, although evidence from standardised studies involving needle inoculation is equivocal [10, 11]. Studies of other haematophagous dipteran groups have also illustrated that both inter- and intraspecific variation in biting pressure occurs within host species [12, 13]. This variation in response reflects a highly complex and often interrelated combination of visual, thermal and semiochemical cues that are used in host-location [14, 15]. Differential responses to hosts have been investigated between humans for *Culicoides impunctatus* Goetghebuer, a nuisance biting species in the UK [16], and for *Culicoides sonorensis* Wirth & Jones feeding on cattle in the USA [17]. Additionally, differences between livestock species have been investigated in France [18] and the Netherlands [19]. To date, however, no systematic assessment of attraction of *Culicoides* to different breeds of livestock have been made.

A wide range of examples of inter-breed differences in ectoparasite numbers and impact on ruminant livestock have been documented for arthropod groups including ticks [20, 21], flies [22–24] and mites [25]. A large proportion of studies have been conducted on the horn fly, *Haematobia irritans* L. which is relatively straightforward to accurately record on cattle hosts by observation. Variation in ectoparasite load can occur due to a wide, and usually undefined, range of physical and behavioural differences between breeds, the degree of specialisation of the ectoparasite concerned and differences in husbandry methods used in production. To date, differences in attraction to sheep breeds have not been explored in *Culicoides*, although evidence of preferential feeding sites for species have been documented in Europe on livestock and horses [26–28].

A combination of shearing of sheep in early summer to allow wool regrowth before the BTV vector season and treatment with insect repellents has been anecdotally suggested to lead to reductions in the incidence of clinical signs of BTV in South Africa [29]. This is hypothesised to be a result of the fleece acting as a more substantial mechanical barrier to blood-feeding by *Culicoides* at times of high biting pressure. Studies of host preference have also demonstrated that the biting pressure of *Culicoides* on sheep tends to be significantly lower than that found on larger hosts such as cattle and horses, hence techniques that mitigate against transmission may have a greater impact for this species where the probability of transmission is already marginal [19, 30]. The impact of fleece length has been investigated in the field for fly strike (myiasis) inflicted by blowflies (family Calliphoridae), where clipping of the fleece, tail docking and mulesing have traditionally been used to reduce impact [31, 32]. To date, however, we are not aware of any studies that have examined the influence of fleece length on blood-feeding by adult biting flies on sheep.

The aim of this study was therefore to investigate the influence of breed and shearing on host: vector relationships in the field using a modified design of drop traps developed in previous investigations [33]. Data from these areas will provide an initial assessment of the potential impact of these techniques on mitigating against BTV transmission and also inform modelling approaches through the provision of vector: host ratios. Methods and results produced during this study have been previously published in the form of a PhD thesis [34].

## Methods

### Study site

All studies were carried out at a mixed cattle and sheep farm in Bradfield, Berkshire, UK (51°27'09.40"N, 1°09'41.82"W). The specific field location used fell into a 1 km^2^ cell with dominant habitat type of “broadleaved, mixed and Yew woodland” and adjacent cells dominated by “broadleaved, mixed and Yew woodland”, “improved grassland” and “arable and horticulture”. Meteorological data was collected using an automatic weather station (CR800 data logger, Campbell Scientific, UK) recording average conditions every 15 min throughout sampling periods. Data collected were: air temperature (°C); relative humidity (%); solar intensity (Wm^-2^); wind speed (ms^-1^) and wind direction (°). For data analysis, wind direction was transformed using the ArcTangent2 function in Microsoft Excel as it is a circular variable and therefore wind direction at 0° and 360° represent the same direction.

### *Culicoides* trapping procedures

**Fig. 1:**
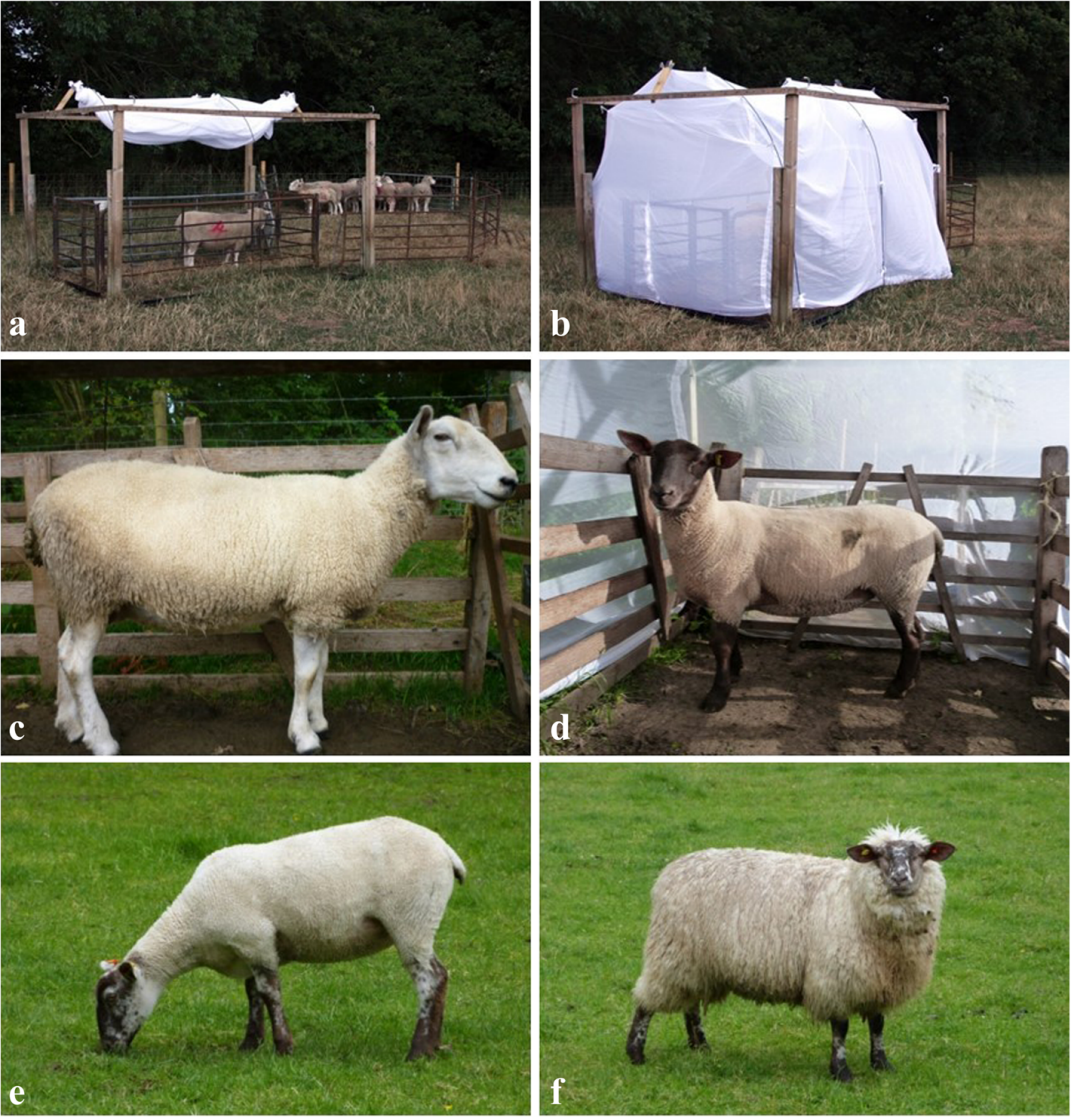
Trapping techniques and hosts used during trials. Panels **a** and **b** show the drop trap design pre- and post-deployment and the connecting corral containing sheep not used in experimentation. Panels **c** and **d** show the Hartline and Hartline/Suffolk cross sheep used in the breed comparison study (Trial 1). Panels **e** and **f** show the sheared and unsheared Hartline/Suffolk sheep (Trial 2)

A drop trap was constructed with a rectangular metal base frame measuring 3 m length by 2.4 m width and three arches were attached to the base frame giving a maximum height of 2.1 m (Fig. 1). The drop trap was further supported by a wooden frame on the outside of the structure. White netting with mesh size of less than 0.25 mm^2^ was attached to the metal frame and could be raised and lowered as required. In order to retain sheep inside the drop trap a rectangular enclosure was created using open sided fencing panels. Prior to *Culicoides* collection, sheep were herded into a corral positioned next to the drop trap where they could be held throughout the sampling period. For each collection sheep were herded into the drop trap with the netting raised. Investigators moved to a distance of at least 100 m from the trap for an exposure period of ten minutes. The investigators then returned, the netting was dropped and all *Culicoides* retained inside the net were collected using a manual aspirator during a 10 min period. *Culicoides* were transferred to pillboxes (Watkins & Doncaster, Leominster, UK), which were then placed in sealed plastic containers with chloroform to kill samples before transfer to 70% ethanol. For each trial a UV light-suction trap was operated as a positive control (Model 912, John W Hock Inc., USA); the trap was positioned at a distance of at least 50 m from the drop trap and two light trap positions were used with the trap switching position each night.

### Trial one: inter-breed variation in *Culicoides* attraction

The trial was conducted from 27/6/2011 to 28/07/2011 [34]. Two breeds of sheep were used: Hartline (a breed originating in the 1970s from a four-way cross of Welsh halfbred/Finn Colbred/Friesland and Texel) and Hartline/Suffolk cross (Fig. 1). Ten females of each breed weighing between 70–80 kg were used and each had a lamb, although collections were only carried out on adults. For each drop trap collection, three individuals of the same breed were used with collections alternating between the two breeds. Collections were carried out from 3 h before sunset to 1 h after; the UV light-suction trap was operated continuously for the 4 h trapping period and hence was not included in the analysis.

### Trial two: collection of *Culicoides* from sheared and unsheared sheep

This trial was conducted from 16/05/2012 to 02/06/2012 in the same field as trial one but the location of the drop trap was moved [34]. Twelve Hartline/Suffolk ewes weighing between 70–80 kg were used in two groups, six were sheared in the week prior to the start of the trial and six remained unsheared. Collections were made using groups of three sheep and conducted from 3 h before sunset to 1 hour after sunset, the UV light-suction trap was examined after each drop trap collection.

### Morphological and molecular identification of *Culicoides*

All collections were initially identified morphologically as *Culicoides* by wing pattern using a stereo-microscope [35, 36]. Female *Culicoides* were identified by observation of the abdomen as unpigmented; pigmented; gravid or blood-fed [37, 38]. For *Culicoides* of the subgenus *Avaritia*, identification of females was carried out using morphology for *Culicoides chiopterus* Meigen (where wing markings are pale and body size is reduced). For *Culicoides obsoletus* Meigen, *Culicoides scoticus* Downes & Kettle and *Culicoides dewulfi* Goetghebuer, a multiplex polymerase chain reaction (PCR) assay was used for differentiation [39].

To extract DNA, *Culicoides* were removed from 70% ethanol storage and allowed to dry for 10 min on paper tissue before being placed individually into 2 ml micro-collection tubes (Qiagen, Manchester, UK). Each tube contained 10 μl of 2% proteinase-k (Bioline, London, UK) in solution with tris calcium acetate and 200 μl of 5% chelex (Bio-Rad, Watford, UK). Samples were homogenised in two cycles of two minutes at 25 Hz in a Tissuelyser (Qiagen), then incubated overnight at 37 °C. 4 μl of each sample was removed and added to a 96 well PCR plate (Abgene, Ashford, UK). These samples were subjected to an 8 min cycle at 99 °C to deactivate the proteinase K.

PCR master mix for each PCR plate consisted: 25 μl of 10 μM forward primer specific to each species (*C. obsoletus*: 5'-TGC AGG AGC TTC TGT AGA TTT G-3'; *C. scoticus*: 5'-ACC GGC ATA ACT TTT GAT CG-3'; *C. dewulfi*: 5'-ATA CTA GGA GCG CCC GAC AT-3') [40]; 25 μl DNAse free water; 100 μl of 10 μM universal reverse primer (5'-CAG GTA AAA TTA AAA TAT AAA CTT CTG G-3') [41]; 7 μl MgCl solution; 400 μl Biomix Red solution (Bioline, UK), 6 μl of master mix was added to each 4 μl sample of extracted DNA. In addition to the test samples, each plate also contained 3 positive controls, using DNA extracted from males of each species using spin column methods with the protocol supplied by the manufacturer (Qiagen), and 3 negative controls consisting of 4 μl of DNAse free water. Samples were subjected to PCR with the following profile: initial denaturing step at 94 °C for 4 min; 32 cycles at 94 °C for 30 s, 60 °C for 30 s, 72 °C for 1 min; followed by a final extension step at 72 °C for 5 min. PCR products were examined by electrophoresis using 2% agarose e-gels (Invitrogen, Glasgow, UK) and identified as species according to band position against positive controls.

Due to the size of the data sets generated in the trials it was not practical to identify all individuals by molecular analysis and a sub-sample method was used. For each trial, five nights of sampling were randomly selected and the entire collections from those nights were subjected to molecular analysis. The proportions of species and physiological states generated by the sub-samples in each trial were then applied to the remaining samples from each trial to infer species and physiological state for the other samples from that trial. Any samples that failed to amplify during molecular analysis were excluded from the final estimates.

### Data analysis

Data were analysed using generalised linear models (GLM) assuming a negative binomial distribution and a log link function in R version 2.15.2 [42]. Models were generated for total numbers of females collected per species and separately for physiological state; all analyses included meteorological variables [34]. Initially, each model included air temperature, relative humidity, solar radiation, wind speed and transformed wind direction. Trap type was included along with a linear and a quadratic temporal trend (number of days since the trial began). Final models were generated using stepwise deletion of non-significant (*P* > 0.05) variables with a final model constructed where all variables are significant (*P* < 0.05) (assuming a linear relationship between log catch size and each meteorological variable). The effects of individual factors in the final model were examined using Tukey’s honest significant differences *post-hoc* test to identify significant differences (*P* < 0.05) between factor levels. Because of the large number of comparisons, a second analysis was carried out in which a Bonferroni correction was applied to set the significance level required for inclusion of a factor in the final model (i.e. the *P*-value, 0.05, divided by the number of comparisons). In this case, significance in trial one was set to *P* < 0.0033 and in trial two it was set to *P* < 0.0036. Applying the Bonferroni correction did not result in differences in the final models and, hence, is not included in the results.

## Results

### Trial one: inter-breed variation in *Culicoides* attraction

**Fig. 2:**
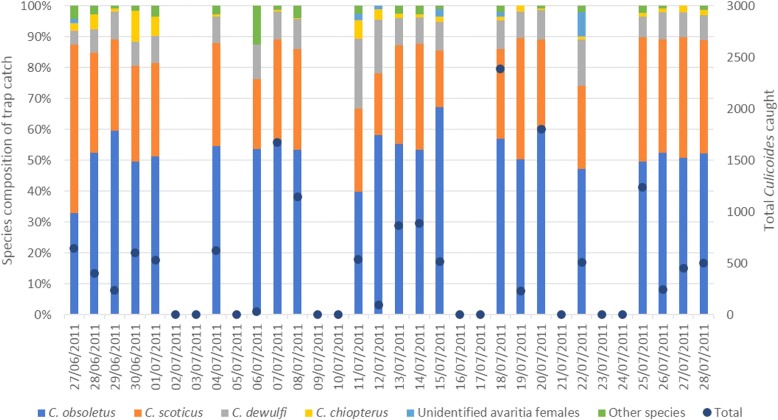
Daily variation in *Culicoides* species composition during Trial 1. Catches were made using drop trap collections made on two different breeds of sheep and from light-suction traps, and total numbers of *Culicoides* caught during each trial day

A total of 224 collections were made from the sheep using the drop trap, 112 from each breed and 22 from the UV light-suction trap (Table 1, Fig. 2). Of 16,151 *Culicoides* collected, 8381 were taken from the cross-breed, 6483 from the pure-breed and 1287 from the light trap. The average *Culicoides* collection for the 10 min exposure period was 74.8 (SEM ± 7.79) for the cross-bred sheep and 57.9 (SEM ± 6.87) for the pure-breed, equating to 24.9 and 19.3 for individual sheep. The largest single collection on the cross-breed was 495 *Culicoides* and for the pure-breed 472, equating to a mean rate on individuals of 165 and 157.3/10 min exposure, respectively. In the light trap the greatest single collection was 498, but this was recorded for the entire four-hour trapping period. Over the course of the trial there were only two collections on sheep where zero *Culicoides* were collected, one for each breed; there were no zero collections made with the light-suction trap.

**Table 1:** Total numbers of *Culicoides* spp. collected using drop trap sampling on two breeds of sheep and from light-suction trap controls (Trial 1)

Trap	Genus/Subgenus/Species					
	Total *Culicoides* spp.	Subgenus *Avaritia* females	*C. obsoletus* males	*C. scoticus* males	*C. dewulfi* males	*C. chiopterus* males
Cross-breed (*n* = 112)	8381	8247	20	7	1	1
Pure-breed (*n* = 112)	6483	6364	34	10	1	1
Light-suction trap (*n* = 22)	1287	1103	7	19	4	1
Total	16,151	15,714	61	36	6	3

Of the *Culicoides* collected, 97.3% were females of the subgenus *Avaritia* (Table 1). Other *Culicoides* species constituted 2.7% of catches: *Culicoides achrayi* Kettle & Lawson (1.3%), *Culicoides punctatus* Meigen (0.5%) and the remaining 0.9% comprised rarer species including *Culicoides pulicaris* L., *Culicoides brunnicans* Edwards and *C. impunctatus*. A total of 2572 individuals identified morphologically as *C. obsoletus*, *C. scoticus* or *C. dewulfi* were subjected to molecular identification through multiplex PCR. Of these, 2458 (95.6%) were successfully amplified and the remaining 114 (4.4%) failed to amplify as a result of poor DNA extraction. Of the successfully amplified samples, 1268 (51.6%) were *C. obsoletus*, 936 (38.1%) were *C. scoticus* and 254 (10.3%) were *C. dewulfi*. Total numbers for these species and physiological states were calculated based on these sub-sample proportions (Table 2).

**Table 2:** Abundance of *Culicoides* spp. of the subgenus *Avaritia* and physiological states calculated from sub-samples of total collections during Trial One

Species	Physiological status	Cross-breed	Pure-breed	Light-suction trap	Total
*C. obsoletus*	Unpigmented	1672	1612	322	3606
	Pigmented	1714	1482	152	3348
	Blood-fed	700	862	6	1568
	Gravid	30	12	46	88
	Male	20	34	7	61
	Total	4136	4002	533	8671
*C. scoticus*	Unpigmented	870	710	283	1863
	Pigmented	377	407	137	921
	Blood-fed	1778	570	5	2353
	Gravid	15	35	18	68
	Male	7	10	19	36
	Total	3047	1732	462	5241
*C. dewulfi*	Unpigmented	368	217	22	607
	Pigmented	446	205	17	668
	Blood-fed	102	112	0	214
	Gravid	5	0	9	14
	Male	1	1	4	6
	Total	922	535	52	1509
*C. chiopterus*	Unpigmented	2	1	3	6
	Pigmented	73	67	19	159
	Blood-fed	63	52	0	115
	Gravid	1	0	3	4
	Male	1	1	1	3
	Total	140	121	26	287
Total		8245	6390	1073	15,708

Statistical analyses of collections on the two breeds of sheep were restricted to investigating differences between the breeds without the direct inclusion of data from the light-suction trap and therefore Tukey’s testing was not necessary as differences between collections between the two breeds are shown in the models. Four models per species were generated to describe the collections of *C. obsoletus*, *C. scoticus* and *C. dewulfi* females from the two breeds of sheep: total females (includes all physiological states); unpigmented females, pigmented females and blood-fed females (Additional file 1: Tables S1-S3). Due to low numbers of unpigmented *C. chiopterus* females collected only three models were generated for this species: total *C. chiopterus* females; pigmented females; and blood-fed females.

Summary statistics for models produced are provided in Additional file 1. Across all four models generated to describe collections of *C. obsoletus* and the three models for *C. chiopterus*, the analyses revealed that catches on the pure- and cross-breeds did not differ significantly (*P* > 0.05) (Additional file 1: Tables S1 and S4). Analysis of *C. scoticus* data revealed that collections differed significantly between the two breeds when considering total females and blood-fed females (*P* < 0.001), but that no significant differences were found for unpigmented or pigmented individuals (Additional file 1: Table S2). Collections of *C. dewulfi* differed significantly between breeds (*P* < 0.001) with greater collections made on the cross-breed, with the exception of blood-fed females where no significant difference was found (*P* > 0.05) (Additional file 1: Table S3).

Temporal trends were significant across all four species (*P* < 0.05). Of the meteorological variables included, wind speed was highly significant in models of all species and physiological states (*P* < 0.001), with reduced catches associated with higher wind speeds. The relationships between trap catches and other meteorological variables were not significant (*P* > 0.05) for all species and physiological states, but where they were significant, the effect was consistent (Additional file 1: Tables S1-S4). Higher trap catches were associated with increased temperatures, with increased humidity and with decreased solar radiation.

### Trial two: collection of *Culicoides* from sheared and unsheared sheep

**Fig. 3:**
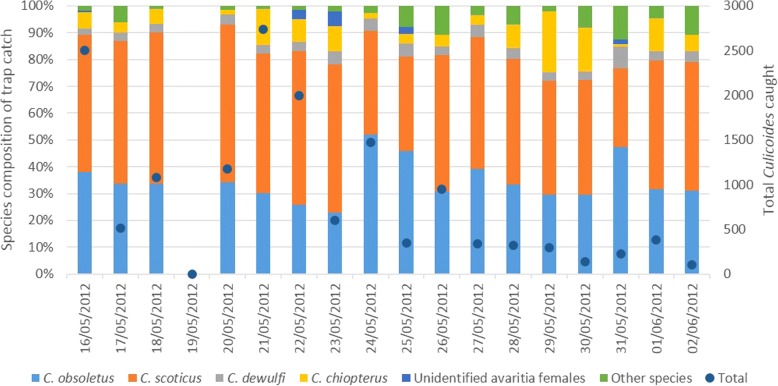
Daily variation in *Culicoides* species composition during Trial 2. *Culicoides* from drop trap collections made on sheared and unsheared sheep and from light-suction traps, and total numbers of *Culicoides* caught during each trial day

A total of 362 collections were made during the trial, 181 in the UV light-suction trap, 90 on the unsheared sheep and 91 on the sheared sheep over 17 nights of sampling (Table 3, Fig. 3). The total number of *Culicoides* collected was 15,163, including 14,613 (96.4%) subgenus *Avaritia* females. Other species collected included *C. brunnicans* (1.92%); *C. achrayi* (0.5%) and *C. pulicaris* (0.4%), while *C. punctatus* and *C. impunctatus* were collected in smaller numbers. The average *Culicoides* collection for the 10 min exposure period was 83.2 (SEM ± 11.45) on the sheared sheep, 75.06 (SEM ± 11.26) on the unsheared sheep and 4.62 (SEM ± 3.29) for the UV light-suction trap. The largest single drop trap collection on the sheared sheep was 523, while the drop trap with the unsheared sheep collected a maximum of 505 individuals. The largest UV light-suction trap collection was 586 *Culicoides*. Assuming all these *Culicoides* fed successfully, this equated to 17.43 bites/min on unsheared individuals and 16.83 for sheared. Over the course of the trial there were only two collections on sheep where zero *Culicoides* were collected, one for each type of sheep, compared to 160 zero samples when using the light-suction trap.

**Table 3:** Total numbers of *Culicoides* spp. collected on sheared and unsheared sheep and with a UV light-suction trap (Trial 2)

Trap type	Genus/Subgenus/Species					
	Total *Culicoides*	Subgenus *Avaritia* females	*C. obsoletus* males	*C. scoticus* males	*C. dewulfi* males	*C. chiopterus* males
Sheared (*n* = 91)	7571	7239	24	4	2	15
Unsheared (*n* = 90)	6755	6565	26	3	8	13
Light-suction trap (*n* = 181)	837	809	2	0	0	0
Total	15,163	14,613	52	7	10	28

Within the subgenus *Avaritia*, 1080 individuals were classified morphologically as female *C. chiopterus*. Of the remaining *C. obsoletus*, *C. scoticus* and *C. dewulfi*, 5050 individuals were subjected to molecular identification as a subsample. A total of 4915 individuals, representing 97.3%, were successfully identified through PCR, the remaining 135 (2.67%) failed to amplify. The results of the PCR revealed that the sub-sample comprised 1824 (37.1%) *C. obsoletus*, 2903 (59.67%) *C. scoticus* and 188 (3.36%) *C. dewulfi*. For each treatment the proportion of physiological state per species calculated from the sub-sample was then applied to the remaining samples to impute total numbers and physiological states for each species (Table 4). For *C. obsoletus* and *C. scoticus* four models were generated to describe collections made on sheared and unsheared sheep: total females; unpigmented females; pigmented females; and blood-fed females (Additional file 1: Tables S5-S11). For *C. dewulfi* insufficient blood-fed individuals were collected for analysis and this was also the case for unpigmented females in *C. chiopterus*.

**Table 4:** Imputed abundance of *Culicoides* spp. of the subgenus *Avaritia* and physiological states calculated from sub-samples of total collections from trial two

Species	Physiological status	Sheared	Unsheared	Light trap	Total
*C. obsoletus*	Unpigmented	1224	1456	383	3063
	Pigmented	356	501	169	1026
	Blood-fed	816	173	3	992
	Gravid	24	7	47	78
	Male	24	26	2	52
	Total	2444	2163	604	5211
*C. scoticus*	Unpigmented	2667	2503	114	5284
	Pigmented	788	597	22	1407
	Blood-fed	525	482	0	1007
	Gravid	0	0	18	18
	Male	4	3	0	7
	Total	3984	3585	154	7723
*C. dewulfi*	Unpigmented	146	184	27	357
	Pigmented	51	35	15	101
	Blood-fed	18	21	0	39
	Gravid	7	13	6	26
	Male	2	8	0	10
	Total	224	261	48	533
*C. chiopterus*	Unpigmented	14	14	0	28
	Pigmented	312	304	0	616
	Blood-fed	226	207	0	433
	Gravid	1	2	0	3
	Male	15	13	0	28
	Total	568	540	0	1108
Total		7220	6549	806	14,575

Summary statistics for models produced are provided in Additional file 1. No significant differences were observed in the total female models between sheared and unsheared sheep, unpigmented models or pigmented models for *C. obsoletus*, *C. scoticus*, *C. dewulfi* or *C. chiopterus* (Additional file 1: Tables S5-S11). In *C. obsoletus*, however, collections using the sheared sheep collected significantly higher numbers of blood-fed individuals than the unsheared (*P* < 0.001), though no such difference was observed in *C. scoticus*, *C. dewulfi* or *C. chiopterus* (Additional file 1: Tables S5-S11).

Solar radiation, wind speed and temperature had a significant impact on catches, with higher trap catches associated with lower solar radiation, lower wind speeds and increased temperatures (Additional file 1: Tables S5, S7, S9 and S11) alongside significant temporal trends in abundance (*P* < 0.05). Light-suction trap collections were also significantly smaller than collections on sheep across all models and light trap position also influenced catches (*P* < 0.05).

## Discussion

This study clarifies several aspects of *Culicoides*: host interactions that to date have been only poorly explored and emphasises species specific responses that may influence arbovirus epidemiology. Biting rates recorded were more than five times higher than those found in an experiment previously conducted in the UK [33]. The *Culicoides* fauna at the site was dominated by the BTV and SBV vectors *C. obsoletus* and *C. scoticus*, although *C. chiopterus* was also found in significant numbers without being caught in high abundance at light, an underestimation that is thought to result from diurnal activity [19, 43]. *Culicoides pulicaris* and *C. punctatus*, despite being implicated as putative vectors of both SBV [44, 45] and BTV [46] were only present in very small numbers on sheep, again in agreement with previous studies in northern Europe [30, 33]. The response to meteorological conditions were also consistent with previous studies that highlighted peak *Culicoides* activity under low wind speeds (< 3 mps^-1^), warm temperatures (15–25 °C) and low solar intensity (< 200 Wm^-2^) [33].

The two sheep breeds selected for the trial were purposefully closely related and of very similar size to reduce the diversity of potential cues used for differentiation. Despite this, breed-specific responses were recorded for both *C. scoticus* and *C. dewulfi*, which have been implicated in transmission of both BTV [46, 47] and SBV [44]. The use of three randomly selected sheep in trials in each replicate was designed to minimise intra-breed variation due to physiological status or physical differences. The biological reasons for the differences found in collections between breeds are unknown. Using silhouettes of cattle and a range of semiochemicals, it has been proposed that visual cues play an important initial role in host location for *Culicoides brevitarsis* [48]. One possible explanation is that greater numbers of *Culicoides* were attracted to the Hartline/Suffolk crosses due to their black facial pigmentation (Fig. 1). A study on *C. sanguisuga* in the USA found that collections on darker coloured hosts were higher than those on lighter hosts [49], although these differences were not statistically significant. Paired trials where visual cues could be isolated from other olfactory cues would therefore be informative in isolating factors that might influence attraction [50].

The effect of shearing on the biting and successful feeding rate of *Culicoides* on sheep had also not been quantitatively investigated prior to this study. Before shearing, the sheep used possessed an extremely thick fleece of 6–8 cm length which covered the majority of the body surface and potentially acted as a barrier to *Culicoides* bites in certain areas of the body (particularly the belly). It was hypothesised that the presence of the fleece would decrease biting rates due to this mechanical barrier, but that there could also have been a secondary effect on attraction of *Culicoides* to the host through the emission of greater quantities of semiochemicals from the fleece or as a by-product of increased respiration due to thermal stress.

While it did not apparently impact on host-location, shearing did enable an increase in successful engorgement of *C. obsoletus*, but not the other three species of the subgenus *Avaritia*. This could relate to differences in feeding regions as *C. chiopterus* has been found primarily (> 90%) to feed on the legs of sheep and cattle [19, 51] and has also been observed to approach sheep hosts at a very low height (S. Carpenter, personal observation). In contrast *C. obsoletus* and *C. obsoletus* complex have been found to land on the belly, back and flanks of sheep and cattle while a recent study in Germany showed no significant differences in landing rates on different parts of sheep although the study did not detail species abundance per body part the predominant species in the collections were *C. obsoletus* complex [19, 26, 27, 52]. These studies show that shearing of sheep would be more likely to impact engorgement of *C. obsoletus* complex than it would *C. chiopterus*. More detailed studies of these differences in behaviour (particularly in *C. scoticus* and *C. dewulfi*) could explain this apparent differential response to shearing and allow prediction of impact on other species. The effect of host coat on feeding behaviour has been investigated in other species of biting flies showing impact of density and length of coat on biting flies it would be logical, therefore, to assume that length of fleece on sheep would affect the feeding capability of *Culicoides* species which feed above the legs [52–54] . Ideally, these studies could use the same sheep for pre- and post-shearing assessments, rather than different cohorts in the current study, reducing the impact of individual variation.

As mitigation against infection with arboviruses, the significant reduction in feeding of *C. obsoletus* on the Hartline breed is of interest given its consistent implication as a vector of BTV. Sheep shearing is usually conducted in June-July in the UK, and this timing often coincides with seasonal peaks in *Culicoides* numbers [55, 56]. The use of earlier or later shearing, informed by both awareness of local transmission of arboviruses and the presence of reservoir hosts, could therefore impact upon infection. This action, however, would need to be balanced with the cost of delaying or bringing forward shearing and the potential for reduction in efficacy of insecticidal treatments on unsheared sheep [57].

The use of groups of three sheep throughout the trial was necessitated by both ethical and experimental concerns surrounding behaviour of sheep when held individually in the pen. Sheep held individually became distressed with increased respiration and additionally moved around in the pen to a far greater degree. While the use of three sheep in each trial would be expected to reduce the impact of individual variation in attractiveness to *Culicoides*, this impact cannot be entirely discounted and future studies would be useful in assessing the impact of this phenomenon within this host species.

Previous studies have produced evidence that traditional means of control such as insecticide application to hosts or larval habitat modification can reduce *Culicoides* host contact rates [7]. There has, however been very limited assessment to date of the impact of such techniques on arbovirus transmission and where trials based on infection have been carried out in the USA and Australia, results have been equivocal [58, 59]. Both of these studies, however, were carried out using cattle with substantial untreated reservoir populations and intense BTV transmission in close proximity. The impact of integrated approaches to *Culicoides* control should therefore be a research priority, particularly for scenarios where the probability of transmission is marginal [8].

## Conclusions

The parameters examined in this study are influential in determining the likelihood of transmission of arboviruses. These data will also play a role in future modelling exercises of arbovirus transmission due to a lack of manipulative studies of the genus and additionally provides an insight into the likely utility of shearing in mitigating arbovirus spread.

## Additional file

Additional file 1: **Table S1.** Regression coefficients for the final negative binomial GLMs for subgenus *Avaritia* female *Culicoides* collected on two breeds of sheep. **Table S2.** Regression coefficients for the final negative binomial GLMs for *C. scoticus* females collected on two breeds of sheep. **Table S3.** Regression coefficients for the final negative binomial GLMs for *C. dewulfi* females collected on two breeds of sheep. **Table S4.** Regression coefficients for the final negative binomial GLMs for *C. chiopterus* females collected on two breeds of sheep. **Table S5.** Regression coefficients for the final negative binomial GLMs for collections of *C. obsoletus* females collected on sheared and unsheared sheep. **Table S6.** Differences in collections between sheared and unsheared sheep and UV suction-light trap controls for *C. obsoletus* for total females (a), unpigmented females (b), pigmented females (c) and blood-fed females (d). **Table S7.** Regression coefficients for the final negative binomial GLMs for collections of *C. scoticus* females from sheared and unsheared sheep. **Table S8.** Differences in collections between sheared and unsheared sheep and UV light-suction trap controls for *C. scoticus* females (a), unpigmented females (b) and pigmented females (c). **Table S9.** Regression coefficients for the final negative binomial GLMs for collections of *C. dewulfi* females from sheared and unsheared sheep. **Table S10.** Differences in catch collections between sheared and unsheared sheep and UV light-suction trap controls for *C. dewulfi* females (a), unpigmented females (b) and pigmented females (c). **Table S11.** Regression coefficients for the final negative binomial GLMs for collections of *C. chiopterus* females on sheared and unsheared sheep. (DOCX 37 kb)
