# Supplementary material for: Sheep breed and shearing influences attraction and blood-feeding behaviour of Culicoides (Diptera: Ceratopogonidae) on a UK farm
Source: Parasit Vectors. 2018 Aug 20;11:473. doi: 10.1186/s13071-018-3003-5 (PMC6102838; doi:10.1186/s13071-018-3003-5)
Supplement: Supplementary file 1 — Table S1. Regression coefficients for the final negative binomial GLMs for subgenus Avaritia female Culicoides collected on two breeds of sheep. Table S2. Regression coefficients for the final negative binomial GLMs for C. scoticus females collected on two breeds of sheep. Table S3. Regression coefficients for the final negative binomial GLMs for C. dewulfi females collected on two breeds of sheep. Table S4. Regression coefficients for the final negative binomial GLMs for C. chiopterus females collected on two breeds of sheep. Table S5. Regression coefficients for the final negative binomial GLMs for collections of C. obsoletus females collected on sheared and unsheared sheep. Table S6. Differences in collections between sheared and unsheared sheep and UV suction-light trap controls for C. obsoletus for total females (a), unpigmented females (b), pigmented females (c) and blood-fed females (d). Table S7. Regression coefficients for the final negative binomial GLMs for collections of C. scoticus females from sheared and unsheared sheep. Table S8. Differences in collections between sheared and unsheared sheep and UV light-suction trap controls for C. scoticus females (a), unpigmented females (b) and pigmented females (c). Table S9. Regression coefficients for the final negative binomial GLMs for collections of C. dewulfi females from sheared and unsheared sheep. Table S10. Differences in catch collections between sheared and unsheared sheep and UV light-suction trap controls for C. dewulfi females (a), unpigmented females (b) and pigmented females (c). Table S11. Regression coefficients for the final negative binomial GLMs for collections of C. chiopterus females on sheared and unsheared sheep. (DOCX 37 kb) [file 13071_2018_3003_MOESM1_ESM.docx]

Additional file 1

Additional file 1: Table S1. Regression coefficients for the final negative binomial GLMs for subgenus Avaritia female *Culicoides* collected on two breeds of sheep (* *P*<0.05, ** *P*<0.01, *** *P*<0.001, NS p>0.05).

| Parameter | *Subgenus Avaritia* Total Females | *C. obsoletus*  Unpigmented | *C. obsoletus* Pigmented | *C. obsoletus* Blood-Fed |
| --- | --- | --- | --- | --- |
| Intercept | 3.971*** | 3.192*** | 0.977* | 0.899 |
| Temporal Trend | | | | |
| Linear | 0.174*** | 0.180*** | 0.179*** | 0.126*** |
| Quadratic | -0.005*** | -0.006*** | -0.005*** | -0.004*** |
| Trap | | | | |
| Cross Breed | Baseline | Baseline | Baseline | Baseline |
| Pure Breed | -0.058 | -0.075 | -0.119 | 0.188 |
| Temperature | NS | NS | NS | 0.096** |
| Humidity | NS | NS | 0.024*** | NS |
| Solar Radiation | -0.004*** | -0.004*** | NS | -0.007*** |
| Wind Speed | -0.659*** | -0.706*** | -0.661*** | -0.574*** |

Additional file 1: Table S2. Regression coefficients for the final negative binomial GLMs for *C. scoticus* females collected on two breeds of sheep (* *P*<0.05, ** *P*<0.01, *** *P*<0.001, NS p>0.05).

| Parameter | Total *C. scoticus* Females | *C. scoticus*  Unpigmented | *C. scoticus* Pigmented | *C. scoticus* Blood-Fed |
| --- | --- | --- | --- | --- |
| Intercept | 4.089*** | 2.675*** | -0.575 | 3.793*** |
| Temporal Trend | | | | |
| Linear | 0.115*** | 0.162*** | 0.168*** | 0.066* |
| Quadratic | -0.003*** | -0.005*** | -0.005*** | -0.002* |
| Trap | | | | |
| Cross Breed | Baseline | Baseline | Baseline | Baseline |
| Pure Breed | -0.569*** | -0.215 | 0.089 | -1.118*** |
| Humidity | NS | NS | 0.026*** | NS |
| Solar Radiation | -0.005*** | -0.004*** | NS | -0.007*** |
| Wind Speed | -0.703*** | -0.729*** | -0.698*** | -0.667*** |

Additional file 1: Table S3. Regression coefficients for the final negative binomial GLMs for *C. dewulfi* females collected on two breeds of sheep (* *P*<0.05, ** *P*<0.01, *** *P*<0.001, NS p>0.05).

| Parameter | Total *C. dewulfi* Females | *C. dewulfi*  Unpigmented | *C. dewulfi* Pigmented | *C. dewulfi* Blood-Fed |
| --- | --- | --- | --- | --- |
| Intercept | 2.218*** | 1.54*** | -0.477 | -4.915 |
| Temporal Trend |  |  |  |  |
| Linear | 0.215*** | 0.196*** | 0.203*** | 0.349*** |
| Quadratic | -0.007*** | -0.006*** | -0.006*** | -0.011*** |
| Trap |  |  |  |  |
| Cross Breed | Baseline | Baseline | Baseline | Baseline |
| Pure Breed | -0.497*** | -0.529*** | -0.809*** | 0.106 |
| Temperature | NS | NS | NS | 0.243*** |
| Humidity | NS | NS | 0.025*** | NS |
| Solar Radiation | -0.004*** | -0.004*** | NS | -0.010*** |
| Wind Speed | -0.705*** | -0.709*** | -0.774*** | -0.488*** |

Additional file 1: Table S4. Regression coefficients for the final negative binomial GLMs for *C. chiopterus* females collected on two breeds of sheep (* *P*<0.05, ** *P*<0.01, *** *P*<0.001, NS p>0.05).

| Parameter | Total *C. chiopterus*  Females | *C. chiopterus* Pigmented | *C. chiopterus*  Blood-Fed |
| --- | --- | --- | --- |
| Intercept | 4.348*** | 4.262*** | 3.755*** |
| Temporal Trend | | | |
| Linear | NS | -0.071*** | NS |
| Quadratic | -0.001*** | NS | -0.001** |
| Trap | | | |
| Cross Breed | Baseline | Baseline | Baseline |
| Pure Breed | -0.031 | 0.135 | -0.203 |
| Humidity | -0.042*** | -0.044*** | -0.048*** |
| Wind Speed | -0.782*** | -0.847*** | -0.665*** |

Additional file 1: Table S5. Regression coefficients for the final negative binomial GLMs for collections of *C. obsoletus* females collected on sheared and unsheared sheep (* *P*<0.05, ** *P*<0.01, *** *P*<0.001, NS p>0.05).

| Parameter | *C. obsoletus* females | *C. obsoletus*  Unpigmented | *C. obsoletus* Pigmented | *C. obsoletus* Blood-Fed |
| --- | --- | --- | --- | --- |
| Intercept | -1.091* | 1.641*** | -0.532 | -2.309** |
| Temporal Trend | | | | |
| Linear | NS | NS | -0.078*** | NS |
| Quadratic | -0.008*** | -0.009*** | NS | -0.007*** |
| Trap | | | | |
| Light Trap 1 | Baseline | Baseline | Baseline | Baseline |
| Light Trap 2 | 0.623* | 0.945** | 1.046** | -0.378 |
| Sheared | 3.409*** | 2.984*** | 2.803*** | 6.231*** |
| Unsheared | 3.295*** | 3.123*** | 3.209*** | 4.728*** |
| Temperature | 0.058* | NS | 0.084** | NS |
| Solar Radiation | -0.008*** | -0.007*** | -0.009*** | -0.005*** |
| Wind Speed | -0.387** | -0.361** | -0.493*** | -0.333** |
| Wind Direction | -0.002* | -0.002* | NS | NS |

##### Additional file 1: Table S6. Differences in collections between sheared and unsheared sheep and UV suction-light trap controls for *C. obsoletus* for total females (a), unpigmented females (b), pigmented females (c) and blood-fed females (d). Estimates are given for factors on the top row relative to factors in the left hand column (* *P*<0.05, ** *P*<0.01, *** *P*<0.001, NS p>0.05).

(a)

| Trap Type | Light Trap  Position 1 | Light Trap  Position 2 | Sheared  Sheep |
| --- | --- | --- | --- |
| Light Trap  Position 2 | -0.62 | - | - |
| Sheared Sheep | -3.41*** | -2.78*** | - |
| Unsheared Sheep | -3.29*** | -2.67*** | 0.11 |

#####

| Trap Type | Light Trap  Position 1 | Light Trap  Position 2 | Sheared  Sheep |
| --- | --- | --- | --- |
| Light Trap  Position 2 | -0.95* | - | - |
| Sheared Sheep | -2.98*** | -2.04*** | - |
| Unsheared Sheep | -3.12*** | -2.18*** | -0.14 |

(b)

| Trap Type | Light Trap  Position 1 | Light Trap  Position 2 | Sheared  Sheep |
| --- | --- | --- | --- |
| Light Trap  Position 2 | -1.05* | - | - |
| Sheared Sheep | -2.80*** | -1.76*** | - |
| Unsheared Sheep | -3.21*** | -2.16*** | -0.40 |

(c)

(d)

| Trap Type | Light Trap  Position 1 | Light Trap  Position 2 | Sheared  Sheep |
| --- | --- | --- | --- |
| Light Trap  Position 2 | 0.38 | - |  |
| Sheared Sheep | -6.23*** | -6.61*** | - |
| Unsheared Sheep | -4.73*** | -5.11*** | 1.50*** |

##### Additional file 1: Table S7. Regression coefficients for the final negative binomial GLMs for collections of *C. scoticus* females from sheared and unsheared sheep (* *P*<0.05, ** *P*<0.01, *** *P*<0.001, NS p>0.05).

| Parameter | *C. scoticus* Females | *C. scoticus*  Unpigmented | *C. scoticus* Pigmented | *C. scoticus*  Blood-Fed |
| --- | --- | --- | --- | --- |
| Intercept | -1.505* | -1.718*** | -2.585*** | 2.421*** |
| Temporal Trend | | | | |
| Quadratic | -0.011*** | -0.014*** | -0.004*** | -0.009*** |
| Trap | | | | |
| Light Trap 1 | Baseline | Baseline | Baseline | Excluded |
| Light Trap 2 | 1.590*** | 2.065*** | 1.918** | Excluded |
| Sheared | 5.611*** | 5.729*** | 5.702*** | 0.028 |
| Unsheared | 5.677*** | 5.867*** | 5.516*** | Baseline |
| Temperature | 0.111*** | 0.098*** | 0.043* | 0.057** |
| Solar Radiation | -0.008*** | -0.007*** | -0.007*** | -0.006*** |
| Wind Speed | -0.424*** | -0.491*** | -0.469*** | -0.236** |

##### Additional file 1: Table S8. Differences in collections between sheared and unsheared sheep and UV light-suction trap controls for *C. scoticus* females (a), unpigmented females (b) and pigmented females (c). Estimates are given for factors on the top row relative to factors in the left hand column (* *P*<0.05, ** *P*<0.01, *** *P*<0.001, NS p>0.05).

(a)

| Trap Type | Light Trap  Position 1 | Light Trap  Position 2 | Sheared |
| --- | --- | --- | --- |
| Light Trap  Position 2 | -1.590*** | - |  |
| Sheared | -5.611*** | -4.021*** | - |
| Unsheared | -5.677*** | -4.087*** | -0.066 |

(b)

| Trap Type | Light Trap  Position 1 | Light Trap  Position 2 | Sheared |
| --- | --- | --- | --- |
| Light Trap  Position 2 | -2.065*** | - |  |
| Sheared | -5.729*** | -3.664*** | - |
| Unsheared | -5.867*** | -3.802*** | 0.864 |

(c)

| Trap Type | Light Trap  Position 1 | Light Trap  Position 2 | Sheared |
| --- | --- | --- | --- |
| Light Trap  Position 2 | -1.918* | - |  |
| Sheared | -5.702*** | -3.784*** | - |
| Unsheared | -5.516*** | -3.598*** | 0.608 |

##### Additional file 1: Table S9. Regression coefficients for the final negative binomial GLMs for collections of *C. dewulfi* females from sheared and unsheared sheep (* *P*<0.05, ** *P*<0.01, *** *P*<0.001, NS p>0.05).

| Parameter | *C. dewulfi*  Females | *C. dewulfi*  Unpigmented | *C. dewulfi*  Pigmented |
| --- | --- | --- | --- |
| Intercept | -1.815*** | -2.358*** | -4.668*** |
| Temporal Trend | | | |
| Quadratic | -0.009*** | -0.016*** | -0.003* |
| Trap | | | |
| Light Trap 1 | Baseline | Baseline | Baseline |
| Light Trap 2 | 1.236** | 1.374* | 1.626* |
| Sheared | 3.286*** | 3.518*** | 3.251*** |
| Unsheared | 3.499*** | 3.675*** | 2.789*** |
| Temperature | 0.069** | 0.088** | 0.121*** |
| Solar Radiation | -0.006*** | -0.006*** | -0.013*** |
| Wind Speed | -0.303* | -0.331* | NS |
| Wind Direction | -0.002* | -0.002* | NS |

##### Additional file 1: Table S10. Differences in catch collections between sheared and unsheared sheep and UV light-suction trap controls for *C. dewulfi* females (a), unpigmented females (b) and pigmented females (c). Estimates are given for factors on the top row relative to factors in the left hand column (* *P*<0.05, ** *P*<0.01, *** *P*<0.001, NS p>0.05).

(a)

| Trap type | Light Trap  Position 1 | Light Trap  Position 2 | Sheared |
| --- | --- | --- | --- |
| Light Trap  Position 2 | -1.236* | - |  |
| Sheared | -3.286*** | -2.050*** | - |
| Unsheared | -3.499*** | -2.263*** | -0.213 |

(b)

| Trap type | Light Trap  Position 1 | Light Trap  Position 2 | Sheared |
| --- | --- | --- | --- |
| Light Trap  Position 2 | -1.374 | - |  |
| Sheared | -3.518*** | -2.144*** | - |
| Unsheared | -3.675*** | -2.301*** | -0.157 |

(c)

| Trap | Light Trap 1 | Light Trap 2 | Sheared |
| --- | --- | --- | --- |
| Light Trap 2 | -1.626 | - |  |
| Sheared | -2.789** | -1.624*** | - |
| Unsheared | -3.251*** | -1.162* | 0.462 |

##### Additional file 1: Table S11. Regression coefficients for the final negative binomial GLMs for collections of *C. chiopterus* females on sheared and unsheared sheep (* *P*<0.05, ** *P*<0.01, *** *P*<0.001, NS p>0.05).

| Parameter | *C. chiopterus*  Females | *C. chiopterus*  Pigmented | *C. chiopterus* Blood-Fed |
| --- | --- | --- | --- |
| Intercept | 2.408*** | 3.331*** | 0.558 |
| Temporal Trend | | | |
| Linear | -0.156*** | -0.121*** | NS |
| Quadratic | NS | NS | -0.010*** |
| Trap | | | |
| Sheared | -0.007 | -0.029 | 0.181 |
| Unsheared | Baseline | Baseline | Baseline |
| Temperature | 0.112*** | NS | 0.136*** |
| Solar Radiation | -0.003** | NS | -0.003** |
| Wind Speed | -0.711*** | -0.813*** | -0.684*** |
| Wind Direction | 0.002* | NS | 0.003* |
